# Non-invasive vagus nerve stimulation in epilepsy patients enhances cooperative behavior in the prisoner’s dilemma task

**DOI:** 10.1038/s41598-022-14237-3

**Published:** 2022-06-17

**Authors:** Carina R. Oehrn, Lena Molitor, Kristina Krause, Hauke Niehaus, Laura Schmidt, Lukas Hakel, Lars Timmermann, Katja Menzler, Susanne Knake, Immo Weber

**Affiliations:** 1 Department of Neurology, Philipps-University Marburg, Marburg, Germany; 2 Center for Mind, Brain and Behavior (CMBB), Philipps-University Marburg, Marburg, Germany; 3 Department of Neurology, Epilepsy Center Hessen, Philipps-University, Marburg, Germany; 4 Department of Psychology, Theoretical Neuroscience Section, Philipps-University Marburg, Marburg, Germany

**Keywords:** Social behaviour, Epilepsy

## Abstract

The vagus nerve constitutes a key link between the autonomic and the central nervous system. Previous studies provide evidence for the impact of vagal activity on distinct cognitive processes including functions related to social cognition. Recent studies in animals and humans show that vagus nerve stimulation is associated with enhanced reward-seeking and dopamine-release in the brain. Social interaction recruits similar brain circuits to reward processing. We hypothesize that vagus nerve stimulation (VNS) boosts rewarding aspects of social behavior and compare the impact of transcutaneous VNS (tVNS) and sham stimulation on social interaction in 19 epilepsy patients in a double-blind pseudo-randomized study with cross-over design. Using a well-established paradigm, i.e., the prisoner’s dilemma, we investigate effects of stimulation on cooperative behavior, as well as interactions of stimulation effects with patient characteristics. A repeated-measures ANOVA and a linear mixed-effects model provide converging evidence that tVNS boosts cooperation. Post-hoc correlations reveal that this effect varies as a function of neuroticism, a personality trait linked to the dopaminergic system. Behavioral modeling indicates that tVNS induces a behavioral starting bias towards cooperation, which is independent of the decision process. This study provides evidence for the causal influence of vagus nerve activity on social interaction.

## Introduction

The vagus nerve is a central part of the gut–brain axis and bi-directionally links the autonomic and the central nervous system^1^. A range of cognitive and emotional processes can influence autonomic processes via the vagus nerve, i.e. by changes in heart rate or respiration^2^. The impact of vagus nerve activity on cognitive processes, i.e., the other direction of information flow, is less well studied (for a review see^3^). Electrical stimulation of the vagus nerve (vagus nerve stimulation, VNS) can be used to study causal effects of vagus nerve activity on functions of the central nervous system. Invasive and non-invasive VNS constitute a common treatment for medication-resistant epilepsy and depression and can be safely applied in humans^3,4^. Non-invasive transcutaneous VNS (tVNS) is commonly administered via the ear and targets the auricular branch of the vagus nerve, primarily afferent fibers projecting to the nucleus tractus solitarius via the main bundle of the vagus nerve (for a review see^3,5^). Evidence from optical stimulation of gut vagal afferents and invasive VNS (also containing efferent activations) in rodents^6,7^ and auricular tVNS in humans^8–10^ provide converging evidence that activation of afferent fibers of the vagus nerve is associated with enhanced reward processing, reinforcement learning and recognition memory. Animal studies indicate that these alterations in behavior are associated with enhanced dopamine release in the midbrain^6,7,11^. This goes in line with evidence from human imaging studies indicating that tVNS is associated with blood-oxygen-level-dependent (BOLD) changes in brain areas involved in reward-processing, such as the dopaminergic midbrain and striatum^12,13^. Based on current evidence, these effects are function-specific, as studies failed to observe general effects, i.e., effects across age groups, the study population and independent of emotional processing, of tVNS on other cognitive functions such as recognition memory^14,15^, implicit learning^16^, conflict processing^17,18^ or response inhibition^19^.

The polyvagal theory represents a bio-behavioral model that relates vagus nerve activity to social interaction^20^. Based on phylogenetic reasoning and anatomical findings of vagus nerve connectivity, it implicates the efferent part of the vagus nerve in the expression of social behaviors, e.g., through its projections to laryngeal, pharyngeal and facial muscles essential for verbal and non-verbal communication. The role of afferent projections of the vagus nerve to the brain for social behavior is not well characterized. tVNS reliably activates the insula and the prefrontal cortex—brain areas involved in social cognition^21^. Rewarding social stimuli, in particular cooperation with other humans and positive social feedback, recruits dopaminergic basal-ganglia-thalamo-cortical circuits similar to non-social rewards, such as the caudate nucleus, the anterior cingulate cortex and the medial orbitofrontal cortex (e.g.,^22–24^). One might thus hypothesize that the stimulation of afferent fibers of the vagus nerve enhances rewards derived from social interactions. On a behavioral level, this might be reflected in prosocial behavior, e.g., cooperation with other humans.

Studies that indirectly assessed vagus nerve activity by means of heart-rate variability (HRV) in humans report that high HRV, and thus parasympathetic tonus, at baseline is predictive for enhanced social engagement and cooperative behavior [e.g.,^25,26^]. A meta-analysis of 16 studies demonstrated that compassion, an important emotional aspect of social interaction positively correlates with HRV^27^. One study using a causal approach investigating the effect of tVNS on prosocial behavior in healthy participants did not find a difference in task performance between tVNS and sham stimulation^28^. However, the study was conducted in participants with high baseline levels of empathy, which might have resulted in a ceiling effect impeding the detection of behavioral effects. Further, the authors argue that stimulation amplitudes during tVNS (0.5 mA) might have been too low to elicit effects on social behavior. However, a recent study demonstrated that tVNS enhanced attention to faces with salient social cues (i.e., direct gaze) using the same stimulation intensity^29^.

Here, we conduct a single-blinded, sham-controlled, counterbalanced cross-over study and assess the causal relationship between vagus nerve activity and social interaction using a well-established task assessing social behavior, the prisoner’s dilemma paradigm^30^. During this computerized task, subjects believe to play live against opponents and try to maximize points by striking a balance between cooperating and competing with different opponents. The outcome of the subject’s choice depends on the opponent’s decision and the paradigm creates a situation where both players acting purely in their own self-interest will result in a suboptimal choice for both. However, mere cooperation is equally not always the most beneficial game strategy. We hypothesize that tVNS biases behavior towards prosocial actions, i.e., cooperation.

Due to restrictions of the European certification of the tVNS device at the time of study, IRB approval could not be obtained for a study in healthy participants. We thus conducted this project in patients with epilepsy, one of the two indications for which CE mark was granted. In order to minimize effects of the pathology on the results, we chose 19 long-standing seizure-free patients with focal epilepsy without macroscopically visible brain lesions, who had never received invasive or non-invasive VNS treatment. We applied auricular tVNS using parameters that have previously been shown to be associated with stimulation of afferent parts of the vagus nerve (for a review see^3,5^) and sham stimulation, while patients performed the task. Based on trial-by-trial choices of participants, we assessed effects of stimulation on cooperative behavior. Further, we assessed the impact of subject characteristics on stimulation effects. We included the big five personality traits into our analysis, i.e., neuroticism, extraversion, openness, agreeableness, and conscientiousness. Recent studies indicate that specific personality traits, in particular neuroticism and extraversion, are associated with social behavior^31^. While personality traits and tVNS-associated cognitive effects are undoubtedly influenced by multiple neurotransmitter systems, specifically extraversion and neuroticism have been linked to dopamine-dependent reward-processing [e.g.,^32,33^]. Thereby, a highly reactive dopaminergic system, e.g., as measured by dopamine-relevant genes, structural volume of dopamine-rich brain regions or dopamine receptor availability, has been associated with high extraversion, whereas the opposite has been suggested for neuroticism. If the effects of tVNS on social behavior in this study are mediated via the dopaminergic system, one could thus hypothesize that individual stimulation effects interact with these personality traits.

To understand the impact of tVNS on social decision making in more detail, we used behavioral modelling. Decision making processes can be disentangled into several sub-processes based on choices and reaction time. Drift–diffusion modelling (DDM) constitutes one of the most common methods for the assessment of value-based choices^34^. DDM dissects the decision process into several sub-processes including a starting bias towards response options, the rate of accumulation of information (i.e., the drift rate), the amount of information needed for a decision (i.e., boundary separation) and non-decision operations reflecting perceptual and motor computations. Previous studies show an association between shifts in starting bias and reward value expectation [e.g.^35–37^]. While the drift–diffusion model is commonly used to make inferences on classic perceptual decision making tasks, only few studies have analyzed sub-components of social decision making in humans^38–41^. These studies report associations between pro-social social behavior and changes in starting bias and drift rate, both relatively early parts of the decision process^40,41^. Thus, we hypothesize that tVNS effects on cooperative behavior occur at these early stages of the decision process.

## Methods

### Participants

In order to estimate effect size of tVNS on social behavior, we first conducted a pilot study with three patients. We subsequently estimated sample size based on the mean and standard deviation of the percentage of cooperations during tVNS and sham stimulation (by means of the function sampsizepwr of the Statistics and Machine Learning Toolbox implemented in Matlab). This analysis indicated that a minimum of 18 participants is required to reveal an effect of stimulation on cooperations (α = 0.05) with 95% power. As our study design entailed the completion of the paradigm on two separate study days with an interval of 14 days between measurements, we expected a 20% drop out rate. We therefore recruited 23 VNS-naïve patients with temporal lobe epilepsy throughout the course of one year. We excluded patients with neurological or psychiatric comorbidities by means of the medical history, the Beck Depression Inventory (BDI-II^42^) and the Quality of Life in Epilepsy questionnaire (QoLiE^43^). To minimize the impact of epilepsy on the results, we exclusively included participants with a minimum seizure-free period of one year and without hippocampal sclerosis. Epileptic medication was stable across testing sessions. Four participants withdrew from the study between testing days, resulting in a final number of 19 participants with complete data sets (13 females; mean age ± SD: 45 ± 12 years). Participants completed a neuropsychological test battery including measures of executive functioning, memory, recall and implicit memory. Further, we obtained self-ratings on the NEO Personality Inventory by Costa und McCrae (NEO-PI) assessing the big five personality traits^44^ and the positive and negative affect schedule (PANAS^45^, Supplemental Table S1). All participants signed written informed consent. The study protocol was approved by the ethics committee of the Faculty of Medicine at the University of Marburg and conducted in accordance with the latest version of the Declaration of Helsinki.

### Sham and vagus nerve stimulation

We applied sham and tVNS in a single-blinded, sham-controlled, randomized cross-over within-subjects design and counterbalanced the order of conditions across patients. Nine patients received tVNS during the first, ten during the second testing session. We applied stimulation on two separate days with an interval of 14 days between measurements. In both conditions, we stimulated participants for two hours before and during the behavioral experiment up to a total of four hours. The experimental protocol was identical on both days. We applied stimulation via the NEMOS® tVNS neurostimulator (Cerbomed GmbH, Erlangen, Germany) using the identical stimulation frequency (25 Hz), duty cycle (50%) and pulse width (30 s) across conditions and amplitudes below the individual pain threshold (mean ± SD amplitude: tVNS: 1.17 ± 0.51 mA, sham: 1.17 ± 0.46 mA).

**Figure 1:**
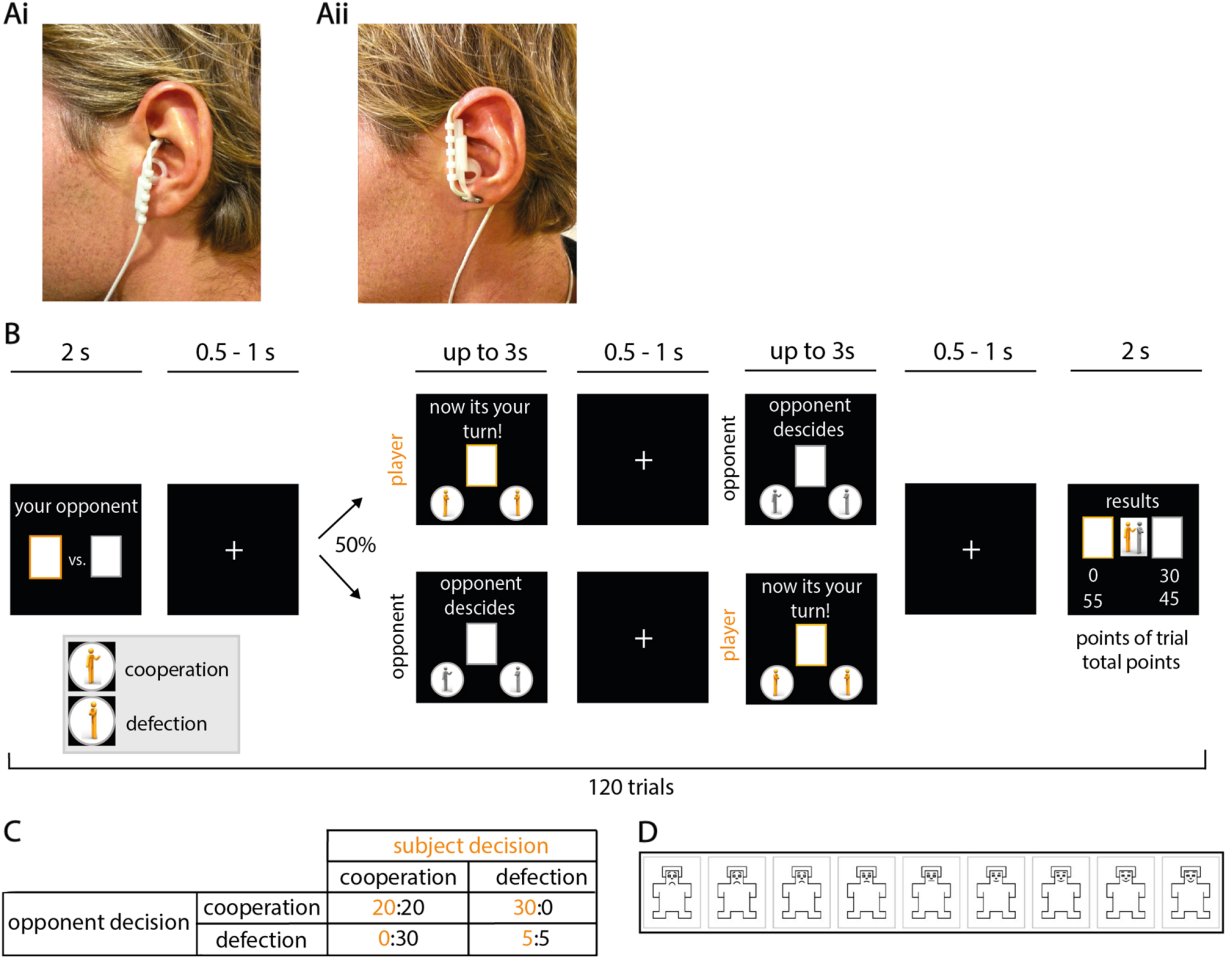
Experimental design and electrode placement. (**A**) The stimulation probe was attached by an independent clinician to the left ear and covered by a headband to conceal stimulation condition to the experimenter. (**Ai**) Following the guidelines of the manufacturer, for the tVNS condition the electrode was applied to the left cymba conchae to stimulate the auricular branch of the vagus nerve. (**Aii**) For the sham condition, the probe was placed in the center of the left lobule (image from^50^). (**B**) Graphical representation of the Prisoner’s dilemma task. (**C**) Points awarded in the prisoner’s dilemma task depending on the players’ and the opponent’s choices. (**D**) Likeability rating of each opponent using the SAM-scale ranging from one (least likeable) to nine (most likeable).

In the active tVNS condition, we applied stimulation to the left cymba conchae to stimulate the auricular branch of the vagus nerve according to the guidelines of the manufacturer (see Fig. 1Ai). This area of the external ear is innervated exclusively by the sensory branch of the vagus nerve, while other parts receive afferent innervation shared with other nerves^46,47^. Current evidence including anatomical and neuroimaging studies, as well as investigations of autonomic parameters in response to auricular tVNS suggests that the cymba conchae constitutes a suitable location for vagal modulation^48^. During sham stimulation, we attached the probe at the center of the left lobule (see Fig. 1Aii). An independent clinician, who was not involved in the acquisition and analysis of data, attached the device on each testing day and subsequently covered the ear using a headband. The location of stimulation was therefore neither visible to the experimenters, nor the participants, who were unaware of the current stimulation condition. Participants were told that the purpose of the study was to test different stimulation settings of tVNS^49^.

### Experimental paradigm

Subjects performed a computerized version of the iterated prisoner’s dilemma paradigm (Fig. 1B^51^) presented on a 24-inch monitor using the PsychToolbox-3^52^ implemented in Matlab (R2016b, Mathworks) in an acoustically shielded chamber. Participants indicated their responses by button press on a standard keyboard. All participants received written and oral instructions and performed several practice trials, in which different stimuli were used than in the subsequent experiment. The experimenter ensured that each participant understood the task before commencing the main part of the experiment.

During the task, subjects played against eight different opponents and could earn points by either cooperating or deceiving their counterpart. The number of awarded points depended on the choice of the player and opponent (Fig. 1C). We instructed subjects to score as many points as possible. To disentangle effects of tVNS on reward-seeking from specific effects on social behavior, we ensured that deception yielded the greatest payoff using predetermined playing strategies of the computerized opponents (maximal possible points per testing day across all opponents: 2175, points achieved when always deceiving: 1725, points achieved when always cooperating: 1500). Subjects completed a total of 120 trials. In 60 trials, subjects were led to believe that they played live against four human opponents (15 trials each). To this end, we implemented a cover story in which the subject played live against university students including delayed logins of one opponent implemented in the Matlab code. In the other half of the trials, subjects knew that they played against four computers (15 trials each). In all trials, subjects played against computers with one of four predetermined game strategies. We assigned the same game strategies to “humans” and computer opponents: cooperative style (70% cooperation), deceitful style (70% deception), tit for tat (replicating the response of the player) and random (drawn from a uniform distribution). Against three of the four opponents, deception represented the most rewarding game strategy, i.e., resulted in the greatest number of points (Supplemental Fig. 1). All trials were presented in a randomized order (across opponents and strategies). To make opponents more relatable to the subjects, each opponent (humans and computers) was introduced in the beginning of the experiment. To this end, we presented images of four humans and computers pseudo-randomly chosen from a pool of 20 human photos of volunteering colleagues and laboratory members (10 women, 10 men) and 10 computer pictures. These images were accompanied by a randomly selected name (from a list of the most common names given between 1985 and 1995), birthplace (selected from a list of medium-sized cities in Germany) and age (between 20 and 30 years) to match the cover story. After introduction of the opponents, we asked subjects to rate the likability of each opponent on a visual rating scale using the Self-Assessment Manikin (SAM^53^, Fig. 1D).

During the experiment, subjects randomly played against each opponent for 15 trials. In each trial, the first screen consisted of a presentation of the opponent. Thereafter, the subject and opponent were successively asked to decide whether they choose to cooperate or deceive each other. The subjects and opponent’s decision were requested in a pseudo-randomized order and the result of both decisions was presented after the second decision had been made. We presented the result alongside the number of achieved points for the last and for all trials. Simulated response times of opponents were randomly drawn from an interval of one to three seconds to match the cover story of live opponents. After completion of all trials, the total number of points gained against each opponent was presented. After both testing days, the experimenters assessed whether subjects believed that they played live against human opponents and whether they had noticed differences between stimulation conditions.

### Statistical analysis

The dependent variable in all our analyses was the percentage of patient’s cooperation in relation to the total number of trials. We performed all calculations with Matlab 2016b (Mathworks), the DMAT toolbox^54^, SPSS (IBM SPSS Statistics, V26) and self-written code. The alpha level for all tests was set to 0.05.

#### Effects of stimulation on cooperation

We discarded trials with invalid responses, i.e., when subjects pressed an undefined key or exceeded the time limit (tVNS: 0–1 trials per subject, sham: 0–6 trials per subject, 16 trials across conditions and subjects). Thereafter, we investigated effects of the independent variables stimulation (tVNS vs. sham) and opponent (human vs. computer) on the number of cooperations (in percent) and reaction times by means of a repeated measures analysis of variance (ANOVA). As control analyses, we further assessed the effect of stimulation on the likeability of opponents, which might indirectly affect cooperation, and on gaming performance measured as total achieved points.

#### Prediction of cooperation

When we found effects in the repeated measures ANOVA, we assessed the impact of several parameters on trial-by-trial task performance by means of a mixed effects logistic regression model. We thereby incorporated several parameters into the model that could impact cooperative behavior either independently or in interaction with the stimulation effect. To this end, we defined the factor “subject” as random effect and assessed fixed effects of opponent characteristics (likability, gaming strategy of opponent, last decision of opponent), subject characteristics (sex, age, NEO-PI scores, believed that they played live against real opponents) and the factor time on the subject’s decision to cooperate or betray. We further incorporated interaction effects between stimulation and all fixed parameters. To avoid multi-collinearity, we first performed a feature selection assuring that predictors were not highly correlated (Pearson correlations, R^2^ > 0.7).

### Behavioral modelling

We dissected the decision process into several cognitive components using DDM. To this end, we included the behavioral parameters “reaction time” and “choice” (cooperation/deception) of all trials and fitted seven nested models for both stimulation conditions separately. For each model, we allowed one model parameter to vary freely for all conditions and compared it to a model with completely fixed parameters using a chi-square difference test at an alpha-level of 0.05^54,55^. The estimated parameters include starting point, drift-rate, non-decision time and boundary separation. Starting point was normalized by the individual boundary separation to improve comparability between individuals and conditions resulting in the starting bias with a range of zero to one with 0.5 indicating no initial preference for either choice. For starting bias and drift-rate, we additionally performed a one-sample t-test against 0.5 and against zero, respectively. We corrected for multiple comparisons by means of Bonferroni correction.

## Results

Our post-experimental questionnaire revealed that none of the patients noticed a difference in stimulation or behavioral performance between the two sessions. Further, patients did not perceive gastrointestinal, cardiac, or other sensations during either stimulation condition. This indicates that patients were unaware of the stimulation condition. Further, patients were not aware of the hypothesis underlying the study including the directionality of expected effects and the different types of stimulation (active vs. sham). All subjects were tVNS-naïve and not familiar with electrode placement for verum and sham stimulation. 12 out of 19 patients believed that they were playing against live opponents.

### Effects of stimulation on cooperations

**Figure 2:**
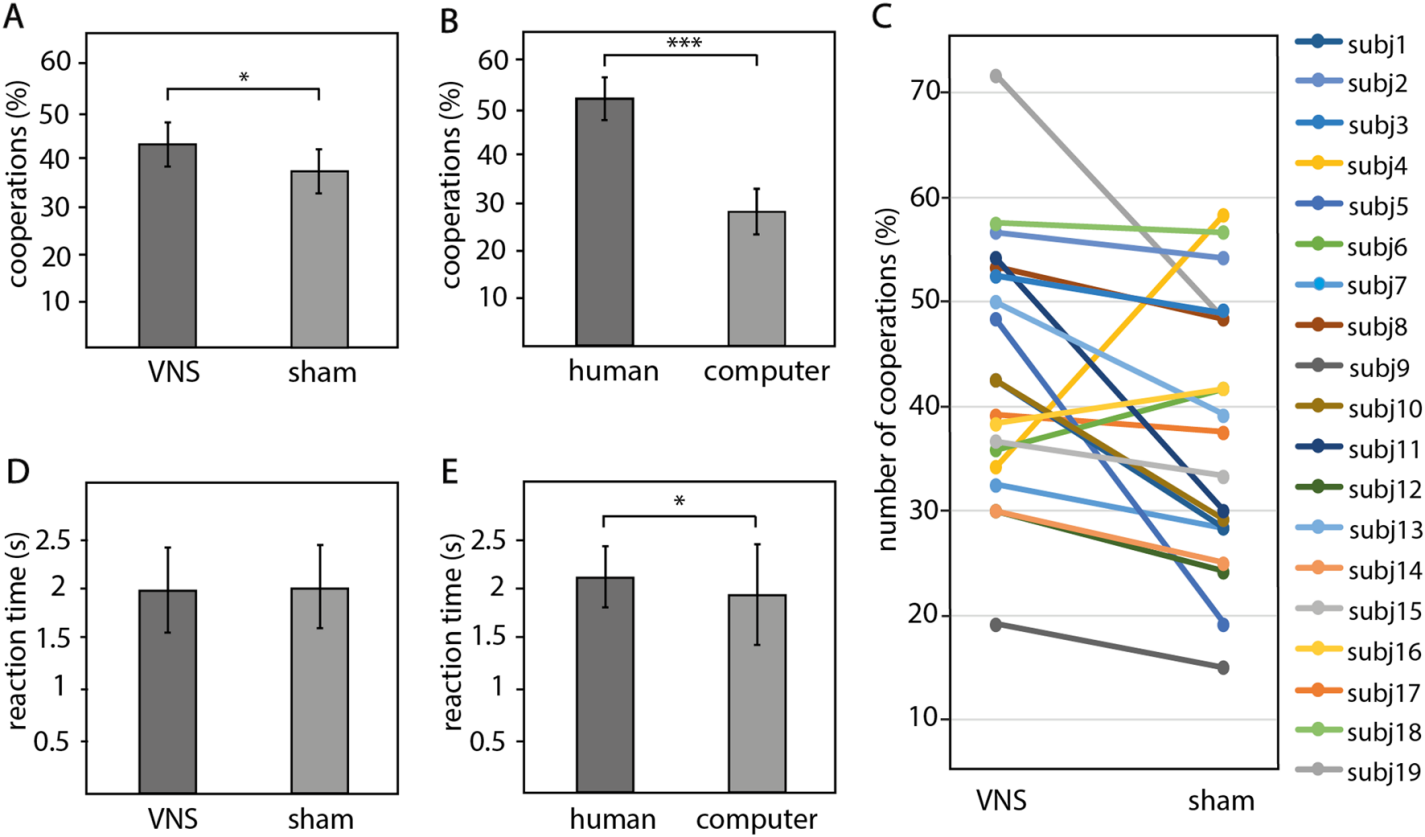
Effect of stimulation on cooperation and reaction times. (**A**–**C**) Average cooperation scores (%) (**A**) for each stimulation condition (tVNS/sham) and (**B**) each opponent (human/computer). Repeated measures ANOVA revealed a main effect of “stimulation” (F_1,18_ = 5.17, p = 0.035) and “opponent” (main effect opponent: F_1,18_ = 24.21, p < 0.001). (**C**) Individual cooperation score (%) for each patient during both stimulation conditions. (**D**, **E**) Average reaction time for each (**D**) stimulation condition and (**E**) opponent. Repeated measures ANOVA demonstrated a main effect “opponent” on reaction times (F_1,18_ = 4.76, p = 0.043). Bar graphs illustrate mean ± SEM parameters across participants.

The repeated measures ANOVA revealed that subjects cooperated more frequently during tVNS compared to sham stimulation (Fig. 2A,C, main effect of stimulation, F_1,18_ = 5.17, p = 0.035; Cohen’s d = 0.52; mean ± standard error of the mean (SEM) number of cooperations: tVNS 43.42 ± 2.80%, sham 37.23 ± 2.87%). Further, they behaved more cooperatively towards humans than computers (Fig. 2B, main effect opponent: F_1,18_ = 24.21, p < 0.001; Cohen’s d = 1.13). The stimulation effect was present for both types of opponents (stimulation*opponent: F_1,18_ = 0.12, p = 0.73; Cohen’s d = 0.08). There were no effects of stimulation on reaction times (Fig. 2D, main effect stimulation: F_1,18_ = 0.08, p = 0.79, Cohen’s d = 0.05; interaction stimulation*opponent: F_1,18_ = 0.09, p = 0.77, Cohen’s d = 0.07). However, players responded slower to human opponents (Fig. 2E, main effect opponent: F_1,18_ = 4.76, p = 0.043, Cohen’s d = 0.50).

As control analyses, we assessed whether stimulation influenced likability ratings and success during the game. There was no effect of stimulation on likeability ratings (main effect stimulation: F_1,18_ = 0.01, p = 0.92, Cohen’s d = 0.02; interaction stimulation*opponent: F_1,18_ = 0.52, p = 0.48, Cohen’s d = 0.16) or total points (main effect stimulation: F_1,18_ = 2.86, p = 0.11, Cohen’s d = 0.39; interaction stimulation*opponent: F_1,18_ = 1.14, p = 0.30, Cohen’s d = 0.24) but a main effect of opponent on likability. Subjects rated humans as more likable (Supplemental Fig. 2A, main effect opponent: F_1,18_ = 54.72, p < 0.001, Cohen’s d = 1.7) and scored less points against human opponents compared to computers (Supplemental Fig. 2B, main effect opponent: F_1,18_ = 5.59, p = 0.03, Cohen’s d = 0.54). Importantly, the stimulation effect did not correlate with clinical parameters (disease duration: R = 0.11, p = 0.65; time since last seizure: R = − 0.22, p = 0.36, affected hemisphere: R = 0.15, p = 0.54 and medication (yes/no): R = 0.05, p = 0.85).

### Prediction of cooperations

All predictors were sufficiently independent (all R^2^ < 0.65). The logistic mixed effects regression model predicting trial-by-trial cooperations revealed main effects of stimulation (t_4514_ = − 2.57, p = 0.01), sex (t_4514_ = 2.96, p < 0.01), likability rating (t_4514_ 5.96, p < 0.001), last response opponent (t_4514_ = 2.33, p = 0.02), extraversion (t_4514_ = 2.29, p = 0.02) and neuroticism (t_4514_ = − 1.98, p = 0.048) on cooperations. Further, we found interaction effects between stimulation and neuroticism (t_4514_ = 4.08, p < 0.001) and extraversion (t_4514_ = − 2.04, p = 0.042). Post-hoc Spearman correlations revealed a decrease of the stimulation effect as a function of neuroticism (Supplemental Fig. 3A, R = − 0.48, p = 0.038), but no correlation with extraversion (Supplemental Fig. 3B, R = 0.23, p = 0.35). Please refer to Supplemental Table S2 for a complete overview of the results.

### Behavioral modelling

**Figure 3:**
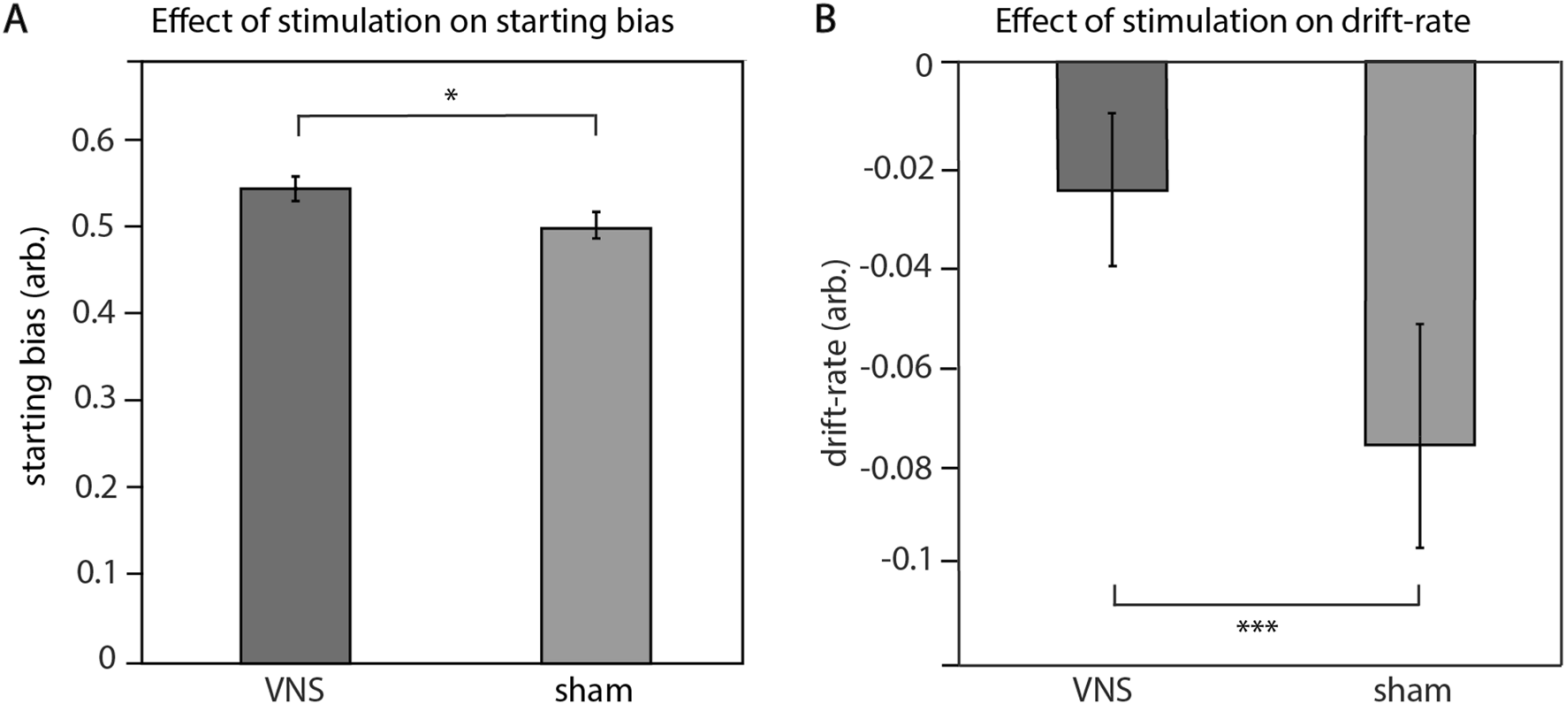
Effect of stimulation on DDM parameters. A chi-square difference test comparing the goodness of fit of the two competing models used in DDM analysis demonstrated stimulation effects on (**A**) starting bias (p = 0.01) and (**B**) drift-rate (p < 0.001). Stimulation increased the starting bias towards cooperation and shifted drift-rate towards zero. Bar graphs illustrate mean ± SEM parameters across participants.

Behavioral modelling revealed that participants expressed a starting bias towards co-operations during tVNS compared to sham (Fig. 3A, p = 0.01, Cohen’s d = 0.54, for fit values see Supplemental Table S3). One-sample t-tests revealed a significant deviation from no starting bias (i.e., 0.5) for the tVNS (p = 0.046), but not the sham condition (p = 1.0). The drift-rate was more positive for tVNS compared to sham stimulation (Fig. 3B, p < 0.001, Cohen’s d = 0.6), with only the sham condition being significantly different from zero (tVNS: p = 0.21, sham: p < 0.01). Further, we found longer non-decision time during tVNS (Supplemental Fig. 4A, p < 0.001, Cohen’s d = 0.05), but no effects of stimulation on boundary separation (Supplemental Fig. 4B, p = 0.1, Cohen’s d = − 0.25).

### Effects of tVNS on mood and other bodily sensations

A paired two sample t-tests revealed no effect of tVNS on acute affect as measured by positive or negative PANAS scores (tVNS vs. sham: positive/tVNS 17.58 ± 1.21 [mean ± SEM], positive/sham 17.74 ± 0.86, p = 0.4; negative/tVNS: 18.2 ± 0.94, negative/sham: 18.58 ± 0.86, p = 0.29).

## Discussion

We assessed the causal relationship between vagus nerve activity and social interaction in humans by means of tVNS. Recent evidence from rodents^6,7^ and humans^8,9^ indicate that tVNS is associated with enhanced reward-seeking and reinforcement learning. To disentangle general effects on reward processing from specific effects on social behavior, we used a paradigm in which deception yielded the greatest payoff. A repeated measures ANOVA and a logistic mixed effects regression model provide converging evidence that tVNS enhanced cooperative behavior compared to sham simulation independent of obtained rewards, i.e., points attained in the game. This indicates that tVNS has a specific effect on social interaction that can be dissociated from general effects on reward-processing. Our logistic mixed effects regression model further revealed that participants cooperated less frequently when opponents had deceived them in the preceding trial and more frequently with opponents they liked, according to the pre-game likability assessment. This emphasizes the impact of social factors on behavior in this computerized task, i.e., that participants did not solely rely on a rational game strategy. Our control analyses demonstrated that tVNS effects were not indirectly mediated by effects on mood and other bodily sensations.

We further investigated the impact of subject characteristics on cooperative behavior and the stimulation effect and found interactions between the effect of tVNS on cooperation and specific personality traits. Our analyses revealed that the stimulation effect decreased as a function of participants’ neuroticism, a personality trait associated with a less functional dopamine system in the brain^33^. Due to our sample size, this result is preliminary and should be interpreted with caution. However, based on recent evidence from rodents suggesting that VNS boots activity in dopaminergic brain regions^6,7^, one could speculate that tVNS exerts a particularly strong effect on social behavior in humans with a less active dopaminergic system. Further, our mixed effects model revealed a positive association between extraversion and the stimulation effect, which, however, was not significant in a post hoc correlation analysis.

To disentangle which sub-components of social decision-making were affected by tVNS, we calculated behavioral models based on choices and reaction times. Behavioral modelling suggests that tVNS influences starting bias, and therefore early stages of the decision process. Human studies demonstrated that the parameter starting point reflects a bias in reward value expectation^35,36^ as well as prior reward probability^36^ independent of the cognitive processing of sensory evidence^37^. These findings indicate that tVNS mediates cooperative behavior by biasing participants’ expectation toward cooperative behavior even before further information about the current opponent is accumulated. Further, we found effects of tVNS on drift rates in line with a previous study showing that a lower absolute drift-rate is associated with pro-social, i.e. altruistic, decision making^40^. Drift rate is thought to reflect the quality of information extracted from the presented stimulus^35,56^. This indicates that after presentation of the opponent, the extraction and accumulation of information leading to cooperative behavior is enhanced. We did find a statistical difference in non-decision time between stimulation conditions, the effect size was close to zero (d = 0.051) and thus its influence on cooperative behavior probably negligible.

One limitation of our study is that we assessed effects in epilepsy patients, as the device had no CE certificate for the use in healthy participants at the time of the study. To reduce possible effects of the disease, we only included patients, who had been seizure-free for at least a year and did not exhibit macroscopically visible lesion in the hippocampus. Further, our control analyses indicate that neither disease duration, time since the last seizure, the affected hemisphere and medication correlated with tVNS effects on cooperation. However, we cannot rule out that microlesions and local changes in neurotransmitter systems impacted the results. Thus, future studies should replicate this finding in healthy controls. Further, it is of interest to assess neural networks involved in the enhancement of cooperative behavior by tVNS. Based on human and animal studies demonstrating effects of VNS on dopaminergic brain circuits and studies showing that social interaction recruits similar networks as reward processing (for a review see^57^), one could speculate the effect of tVNS on social behavior is mediated by dopaminergic neural networks. However, the effect of tVNS on social interaction is likely mediated by a complex process involving alterations in multiple neurotransmitter systems including serotonin and noradrenaline and interactions in brain areas activated by both, tVNS and social behaviors, such as the insula and the prefrontal cortex [e.g.,^21,58^]. The investigation of effect of tVNS on social behavior in healthy participants and the associated neural networks will be subject to future studies. Nevertheless, recent studies indicate that discrepancies between the outcome of studies investigating VNS effects on cognition in epilepsy patients and healthy participants, e.g., on recognition memory^14,59^, are a result of different modes of VNS application, i.e., invasive vs. transcutaneous stimulation respectively, rather than the pathology^15^.

## Conclusion

Taken together, our results indicate that enhanced vagus nerve activity plays a causal role for mediating social interaction and biases participants towards cooperative behavior. This effect is more pronounced in participants with higher scores in neuroticism. Behavioral modelling revealed that the effect of VNS on stimulation occurs at early stages of decision-making, even before stimulus processing. Thus, our results indicate that alterations in vagal tone are not merely an adaptive process in response to social situations, but can also, in return, influence social behavior. The interaction between vagal activity and social behavior is therefore bidirectional.

## Supplementary Information

Supplementary Information.

## Data Availability

The datasets generated and analyzed during the current study and the analysis code are available from the corresponding author upon reasonable request.
