# Supplementary material for: Non-invasive vagus nerve stimulation in epilepsy patients enhances cooperative behavior in the prisoner’s dilemma task
Source: Sci Rep. 2022 Jun 17;12:10255. doi: 10.1038/s41598-022-14237-3 (PMC9205877; doi:10.1038/s41598-022-14237-3)
Supplement: Supplementary file 1 — Supplementary Information. [file 41598_2022_14237_MOESM1_ESM.docx]

**Supplemental Table 1: Overview of patient demographics, results from neurophychological testing and personality traits assessed with the NEO Personality Inventory by Costa und McCrae (NEO-PI).** f: female, m: male; age (years), disease duration: time since diagnosis (years), time since last seizure (years), stimulation amplitude (mA, stimulation amplitude was not different between conditions: two-sided paired t-test: t^18^<0.001, p=1), WAIS-IV: Wechsler Adult Intelligence Scale, MZ_RW: raw values of matrix reasoning test, MZ_WP: point values of matrix reasoning test, NEO-PI scores: neuroticism (N), extraversion (E), openness (O), agreeableness (A) and conscientiousness (C), PANAS: positive and negative affect schedule.

**Supplemental Table 2: Overview of fixed and random effects revealed by the logistic mixed effects regression model.** Stimulation, sex, likeability of the opponent, last response of the opponent, extraversion and neuroticism predicted cooperative behavior. Further, we found interaction effects between stimulation, neuroticism, and extraversion.

**Supplemental Table 3:** Shown are the badness-of-fit values of the DDMs per patient. The lower the values, the better is the model fit.

**
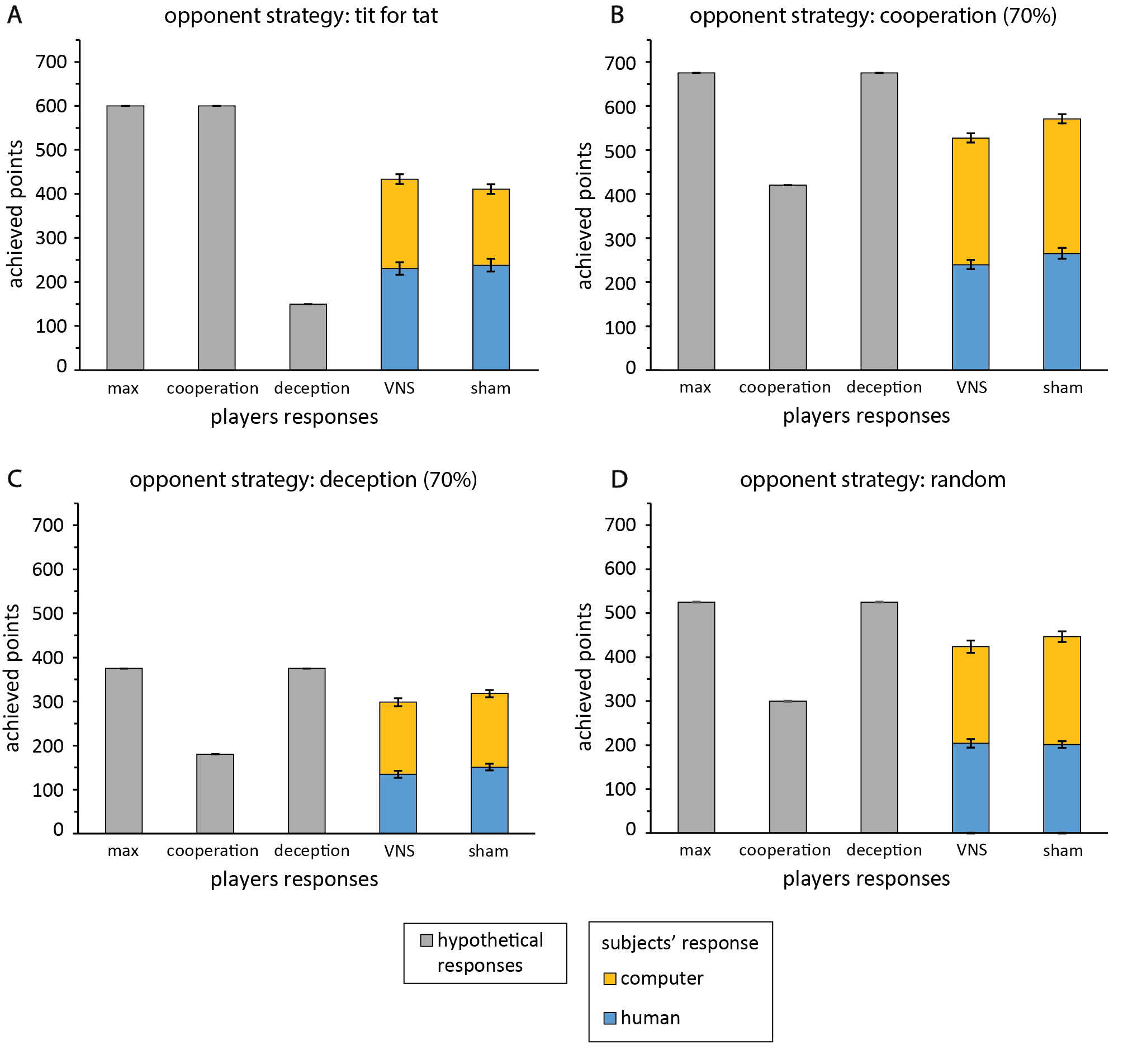
Supplemental Fig. 1: Effect of different game strategies against the four opponents.** Grey bar graphs illustrate a hypothetical number of points that can be achieved per testing day, i.e., one stimulation condition, when playing with the optimal game strategy (max), continuous cooperation or deception. **A)** Playing against the opponent, who uses the ‘tit for tat’ strategy, constant cooperation results in the highest number of points. Against opponents with all other game strategies (**B-D**, cooperation, deception, and random), deception is associated with the highest pay-off. Blue and yellow bar graphs represent participants’ real achieved points against human (blue) and computer opponents (yellow) with the four game strategies during the VNS and the sham testing session.


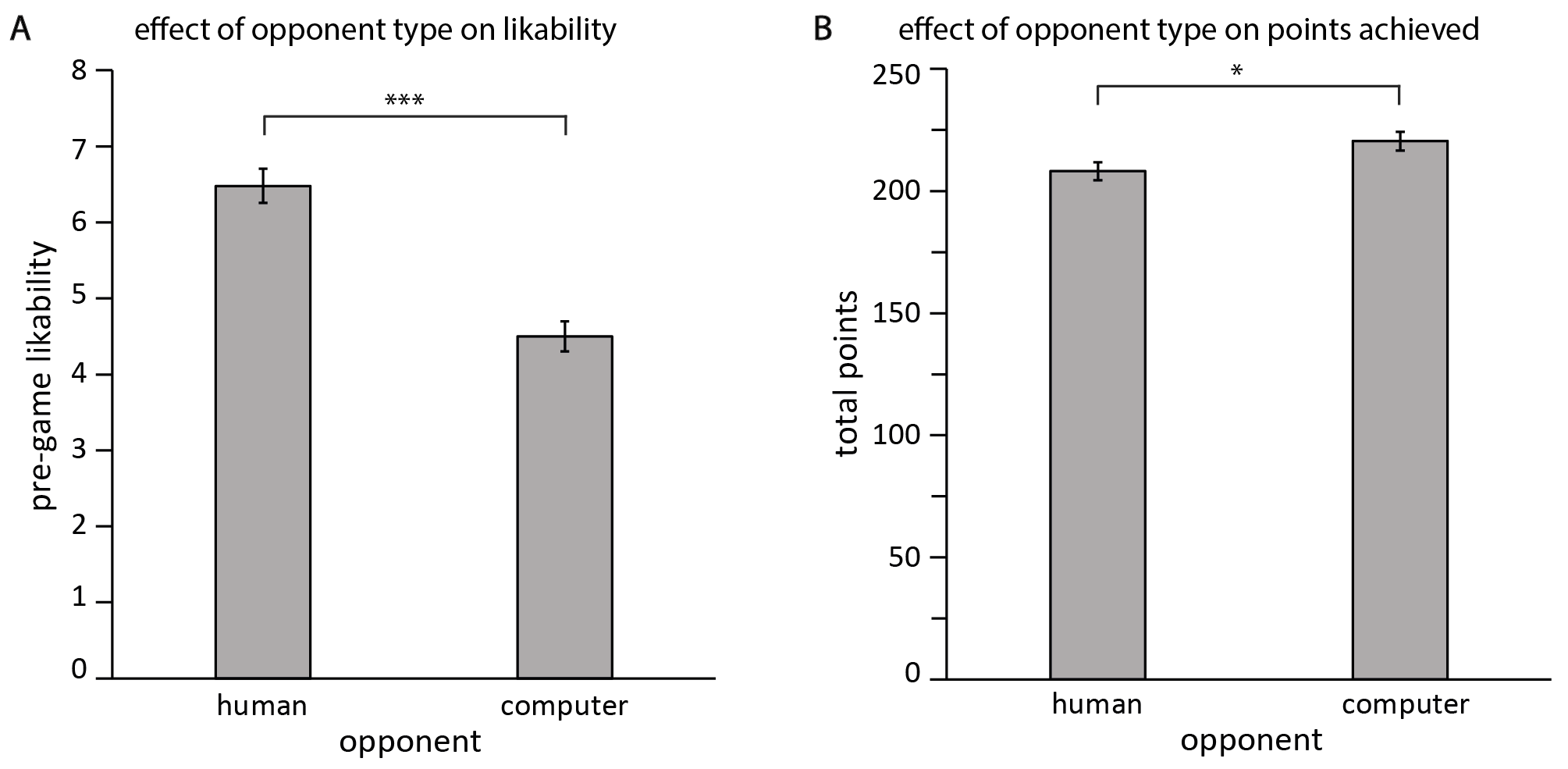
**Supplemental Fig. S2: Main effect of the factor opponent on likability ratings and total points achieved**. **A)** Bar plots indicate mean±standard error of the mean (SEM) likability ratings for human and computer opponents (across characters and stimulation conditions). Subjects rated humans more likable than computers (F_1,18_=54.72, p<0.001). **B)** Bar plots indicate mean±SEM points achieved against human and computer opponents (across characters and stimulation conditions). Participants achieved more points against computers than humans (F_1,18_=5.59, p=0.03).

**
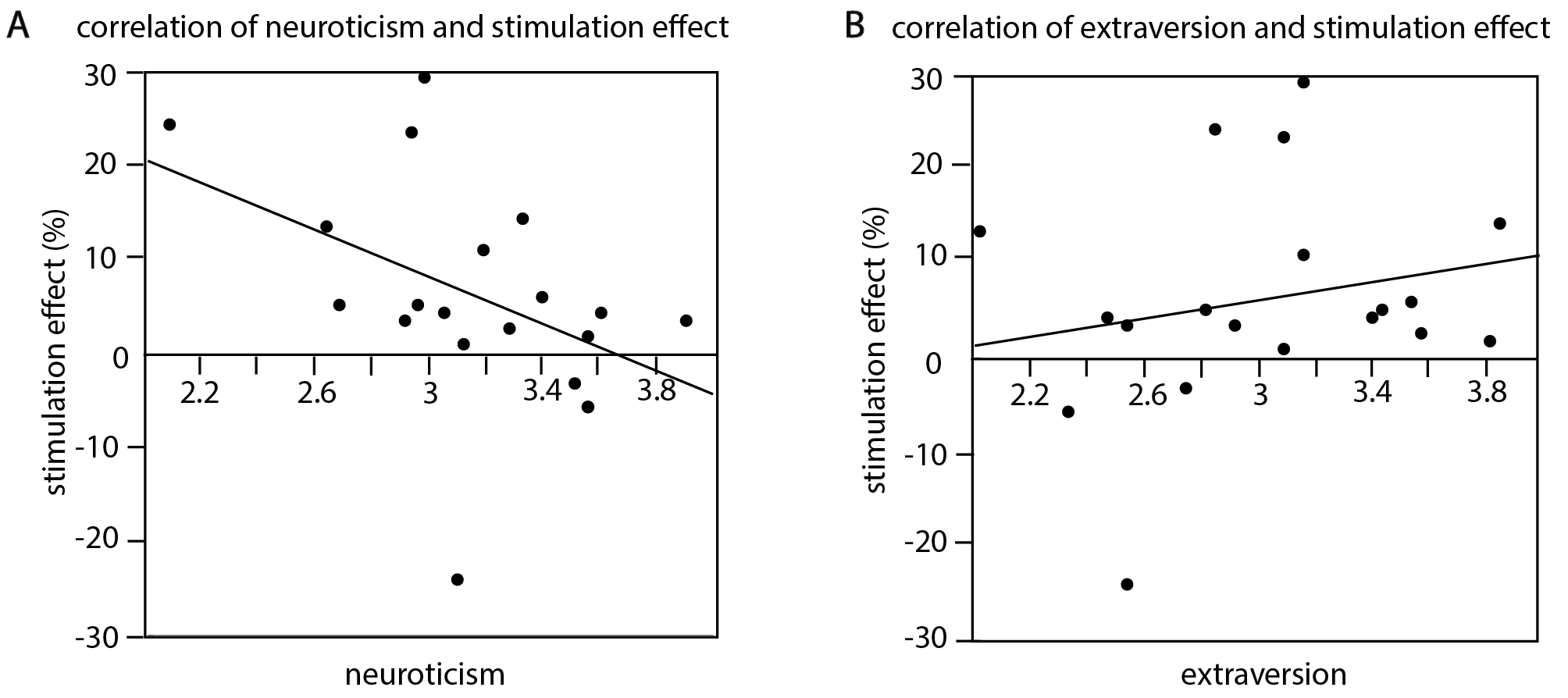
Supplemental Fig. S3: Stimulation effect as a function of neuroticism and extraversion.** Post-hoc Spearman correlations revealed a decreased stimulation effect as a function of **A)** neuroticism (R=-0.48, p=0.038), but not **B)** extraversion (R=0.23, p=0.35). Each point represents the difference between cooperations during the VNS and Sham condition (%) plotted as a function of individual scores of extraversion and neuroticism.

**
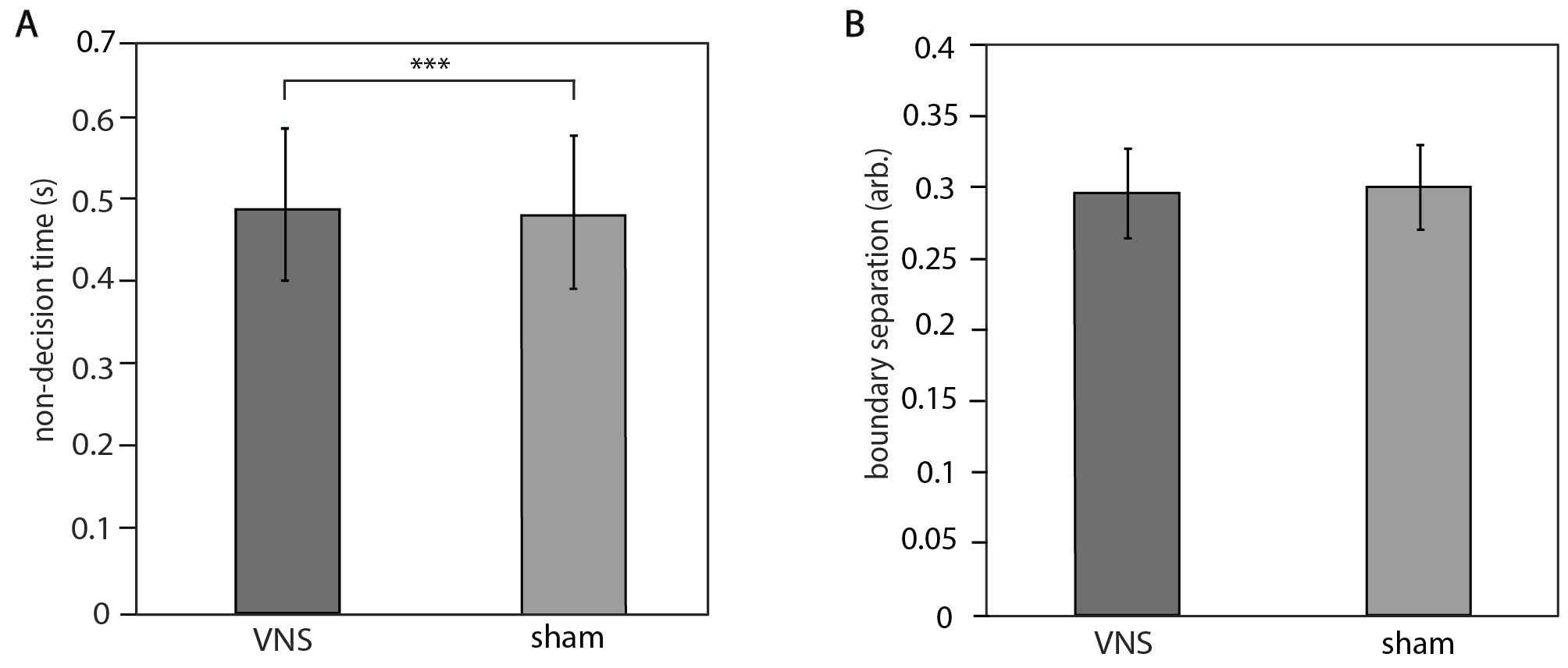
**

**Supplemental Fig. S4: Effect of stimulation on additional DDM parameters.** A chi-square difference test comparing the goodness of fit of the two competing models used in DDM analysis demonstrated stimulation effects on **A)** non-decision time (p<0.001), but not **B)** boundary separation (p=0.18). However, the effect size of the former result was very small (Cohen’s d=0.05).
